# Selected Textural and Electrochemical Properties of Nanocomposite Fillers Based on the Mixture of Rose Clay/Hydroxyapatite/Nanosilica for Cosmetic Applications

**DOI:** 10.3390/molecules28124820

**Published:** 2023-06-16

**Authors:** Victoria Paientko, Olena I. Oranska, Volodymyr M. Gun’ko, Ewa Skwarek

**Affiliations:** 1 Department of Radiochemistry and Environmental Chemistry, Institute of Chemical Sciences, Faculty of Chemistry, Maria Curie-Skłodowska University, 3 Maria Curie-Skłodowska Sq., 20-031 Lublin, Poland; payentkovv@gmail.com; 2 Chuiko Institute of Surface Chemistry, 17 General Naumov Street, 03164 Kyiv, Ukraine; el_oranska@nas.gov.ua (O.I.O.); vlad_gunko@ukr.net (V.M.G.)

**Keywords:** clay minerals, hydroxyapatite, acai, electrical double layer

## Abstract

In order to improve the properties and characteristics of rose clay composites with acai, hydroxyapatite (HA), and nanosilica, the systems were mechanically treated. This treatment provides the preparation of better nanostructured composites with natural and synthetic nanomaterials with improved properties. The materials were characterized using XRD, nitrogen adsorption and desorption, particle sizing, zeta potential, and surface charge density measurements. For the systems tested in the aqueous media, the pH value of the point of zero charge (pH_PZC_) ranges from 8 to 9.9. However, the isoelectric point (pH_IEP_) values for all composites are below pH 2. This large difference between pH_PZC_ and pH_IEP_ is due to the complexity of the electrical double layer (EDL) and the relation of these points to different layers of the EDL. The tested samples as composite/electrolyte solutions are colloidally unstable. The toxicity level of the ingredients and release of anthocyanins as bioactive substances from acai in the composites were determined. The composites demonstrate an enhanced release of anthocyanins. There are some regularities in the characteristics depending on the type of components, morphology, and textural features of solids. The morphological, electrochemical, and structural characteristics of the components have changed in composites. The release of anthocyanins is greater for the composites characterized by minimal confined space effects in comparison with rose clay alone. The morphological, electrochemical, and structural characteristics allow us to expect high efficiency of composites as bioactive systems that are interesting for practical applications in cosmetics.

## 1. Introduction

The development of new delivery forms of bioactive substances (BAS) is an important and urgent problem of modern pharmacology and cosmetology. The use of nanostructured carriers for BAS allows one to obtain dosage forms with improved compatibility with biofluids, controlled time of entry into the body, release kinetics, high storage stability, etc. Creating bionanocomposites providing the necessary content of BAS with the appropriately controlled release is an effective way to obtain positive results in cosmetic applications. The novelty of this study is caused by the selection of composites based on rose clay with acai, hydroxyapatite, and nanosilica not yet described in the literature that may have practical applications in cosmetics.

Clay minerals exhibit interesting properties and characteristics such as appropriate adsorption capacity for BAS, an excellent life cycle for water treatment, effectiveness and remediation potential, as well as relatively low cost and toxicity [1]. Clays are composed of thin lamellar nanoparticles with two-dimensional silicate layers arranged together. The replacement of the inorganic cations in the clays with the organic ones has been extensively studied in order to change the surface properties and characteristics of the clays and to improve their adsorption capacity. These ions reduce the free surface energy of the clays, making them more organophilic, thus facilitating access between the layers. The modification processes can cause significant changes in the surface properties of the clays as well as affect the pore structure and other characteristics. The nanoclays are also known as non-toxic and environmentally friendly materials. These substances have been used as excellent adsorbents [2].

The use of natural or synthetic nanostructured materials allows one to produce nanostructured composites with appropriate control of the physicochemical properties and characteristics including morphological, structural, textural, mechanical, abrasive, thermal, and electrochemical ones. Clays, including rose clay, can be readily modified and mixed with other natural and synthetic materials, e.g., colloidal silica, nanostructured metal oxides, carbons, and bioactive compounds, to be used for medical and cosmetic applications [3,4,5,6,7]. Note that bound water taking part in the hydrogen bond network at the interface of nanostructured solids plays an important role. It strongly affects the properties of nanostructured materials, composites, bioactive materials, etc. [8,9,10,11].

Hydroxyapatite (HA (Ca_10_(PO_4_)_6_(OH)_2_)), is one of the most promising cosmetic and medical biomaterials. It is used as a ceramic material to produce bone implants and coatings of prostheses. This is due to HA’s capability to chemically bind to the bones and chemically resemble a mineral part of hard tissues [12]. However, the properties of hydroxyapatite that can be used in cosmetics largely depend on the additives combined to form nano/microstructurally appropriate composites. A number of techniques can be used to obtain hydroxyapatite: (i) wet methods, including sol-gel, chemical, hydrolytic, hydrothermal, sonochemical, and emulsion precipitation; (ii) dry methods (mechanomechanical and semiconductor); and (iii) synthesis based on biogenic sources and combined procedures [13]. Additionally, hydroxyapatite can be extracted from natural sources (plants and algae) as well as from minerals (limestone), biosources or wastes such as mammal bones (horse, bovine, and camel), marine or aquatic sources (fish bones and scales), and shell sources (tares, mussels, and eggshells) [14].

The characteristics of the interfacial layers between hydroxyapatite and aqueous solutions are important from a practical point of view. Currently, many attempts are devoted to developing biomaterials based on hydroxyapatite [15,16]. The adsorption and adhesive characteristics could be changed by grafted organic molecules having appropriate binding functionalities. Surface modification is intended to strongly improve the performance of the materials in the environment of aggressive body fluids. Hydroxyapatite in contact with liquids, e.g., saliva, in the first stage creates strong bonds with biomolecules that, in turn, cause further interactions with other dissolved substances and cells. The zeta potential (ζ) values (determining some features of these interactions) estimated from the electrophoretic mobility (μ) measurements depend on the chosen equation combining the ζ and μ values that affect the accuracy of obtained results. Additionally, the relationships between ζ and other parameters characterizing the interface—such as the surface potential (ψ_0_) and surface charge density (σ_0_)—depend on the adopted models of the electrical double layer (EDL) structure. There is no universal agreement in this issue [17,18]; however, it is often assumed that ζ = ψ_d_ (potential at the beginning of the EDL diffusion region). Even with these limitations, the electrophoretic mobility measurements provide valid tools for the appropriate characterization of the biomaterial/liquid interfaces. For the EDL, the Gouy–Chapman model could be adopted. The solubility of HA itself can be varied; a part of the surface can have a greater solubility. Additionally, the times reported to reach the solubility equilibrium are quite different. In the absence of foreign ions, hydroxyapatite is the most stable one, i.e., the most insoluble phase in a wide range of solution compositions. The value of the solubility product is logIr = −114 for the following equation:

Ca_10_(PO_4_)_6_(OH)_2(s)_ → 10Ca^2+^ + 6PO_4_^3−^ + 2OH^−^. (1)

When the solution contains other anions such as carbonate or fluoride, or cations, the HA surface can change. Even when ions have a small mass contribution, the surface effects can be significant. Many of the discrepancies in the reported logIr values can be attributed to stoichiometry.

There are few reports in the literature related to the present research [19,20,21,22,23]. This study proposes the creation of composites (vide infra) based on the clay mineral and hydroxyapatite that provide various forms of inclusion of active substances by optimizing the composition and improving the formation methods. Significant hydrophilicity of clays enhances the moisture content in composite materials and, as a consequence, increases the content of bound BAS.

Acai (Euterpe oleraceae (acai) berry) powder was chosen as a source of BAS. Acai berries contain many trace elements such as Ca, P, K, Mg, Zn, B vitamins, beta-carotene, anthocyanins, etc. Some benefits of acai include the following: it moisturizes and nourishes the skin; relieves symptoms of dermatitis, acne, rosacea, and allergic skin manifestations; provides regeneration and rejuvenation of skin cells; has anti-inflammatory action; protects from UV rays; cleanses the skin of toxins to a large depth; improves metabolic processes; and restores and increases skin elasticity [24,25].

## 2. Results and Discussion

### 2.1. Powder XRD Analysis of Rose Clay-Hydroxyapatite-Silica Composites

**Figure 1:**
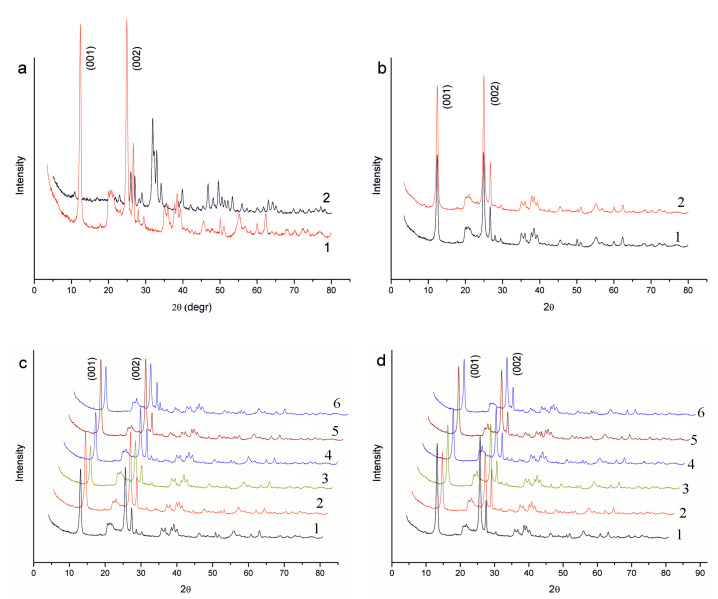
XRD patterns of (**a**) initial components of composites: natural rose clay (1), hydroxyapatite (HA) (2); (**b**) natural rose clay (1) and after its thermal treatment after heating at 100 °C (2); (**c**) composites 1: rose clay (100%, native); 2: rose clay native (95%), hydroxyapatite (5%); 3: rose clay native (97%), silica (3%); 4: rose clay native (92%), hydroxyapatite (5%), silica (3%); 5: rose clay native (90%), hydroxyapatite (10%); 6: rose clay native (87%), hydroxyapatite (10%), silica (3%); and (**d**) composites after the thermal treatment at 100 °C: 1: rose clay (100%); 2: rose clay (95%), hydroxyapatite (5%); 3: rose clay (97%), silica (3%); 4: rose clay (92%), hydroxyapatite (5%), silica (3%); 5: rose clay (90%), hydroxyapatite (10%); and 6: rose clay (87%), hydroxyapatite (10%), silica (3%).

The diffractograms of the initial components (rose clay and hydroxyapatite) are shown in Figure 1a. Kaolinite (Al_4_[Si_4_O_10_](OH)_8_) (ICDD #78-2110) and quartz (SiO_2_) (ICDD #85–1054) are identified as components of rose clay. The hydroxyapatite diffractogram corresponds to a pure phase of HA (ICDD #74–566). The diffractograms of rose clay-hydroxyapatite-silica composites are presented in Figure 1b.

Reflections of the crystalline phases of rose clay and HA are observed on the diffractograms of the samples with the corresponding composition. Based on the analysis of diffractograms, it can be seen that the intensity of peaks of HA increases with an increase in its content in the composites. This can be clearly seen in the diffractograms of samples without silica (curves 2 and 5). The intensity of the peaks of kaolinite and quartz, with the exception of basal reflections of kaolinite, naturally decreases with a decrease in the content of rose clay in the composites. The intensity of basal kaolinite reflections in composites containing only rose clay and HA changes slightly. The inclusion of amorphous silica in composites leads to a noticeable decrease in the intensity of basal reflections of kaolinite. This effect is most pronounced for the sample (curve 3) not containing HA. Thus, the HA internal structure of the components is preserved in the composites. The introduction of silica contributes to the disorientation of particles containing the layered material—rose clay. The diffractograms of rose clay before and after the thermal treatment are shown in Figure 1c. To increase the intensity of basal reflections of heat-treated clay, it is clear that its layered structure improves or, in other words, the orientation capability of the kaolinite particles layers improves.

For the composites prepared using heat-treated rose clay, changes in the diffractograms remain the same as for the original composites. However, in general, the intensity of the basal reflections of rose clay for these samples is higher than that of those for the composites with natural clay. Therefore, such composites have more orientation capability of the layered particles than the original composites (Figure 1d).

### 2.2. Textural Characteristics

**Figure 2:**
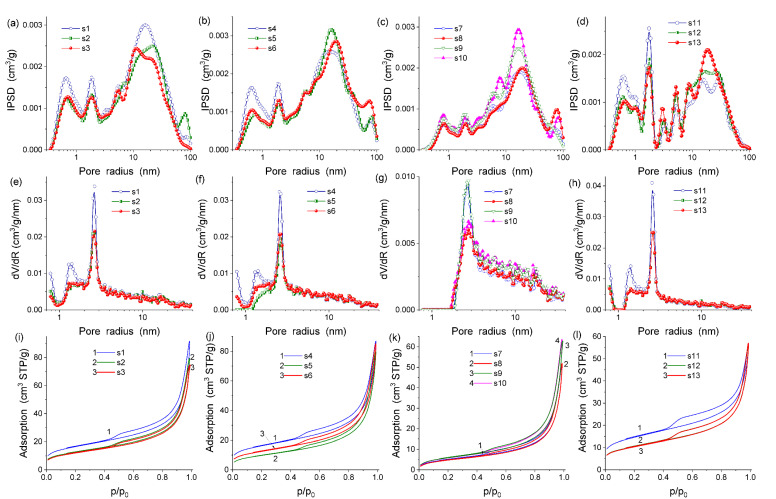
Pore size distributions: (**a**–**d**) incremental with the SCV/SCR method and (**e**–**h**) differential NLDFT, and (**i**–**l**) nitrogen adsorption–desorption isotherms for the samples (**a**,**e**,**i**) s1–s3, (**b**,**f**,**j**) s4–s6, (**c**,**g**,**k**) s7–s10, and (**d**,**h**,**l**) s11–s13.

The textural characteristics (Table 1 and Figure 2) are mainly determined by the main phase with clay in the composites (see Figure 2i–l showing that the isotherm shapes for the composites are similar to that of clay alone). Additionally, the contribution of the slit-shaped pores, characteristic of such clays as kaolinite (a main component of rose clay), is predominant (Table 1, *c*_slit_ > *c*_cyl_ + *c*_sph_).

The sample preheating at 100 °C or 200 °C results in a diminution of the PSD intensity (mainly of narrow pores) and specific surface area (Table 2, *S_BET_* and *S*_NLDFT_), especially for *S*_nano_, because it is smaller for *S*_meso_ and absent for *S*_macro_. The preheating effect for the pore volume (Table 1, *V*_p_) and its components (*V*_nano_, *V*_meso_, and *V*_macro_) depends on the kind of materials.

The values of <*R*_V_> and <*R*_S_> (Table 1) correspond to the main peaks of IPSD SCV/SCR and NLDFT PSD (Figure 2a–h), respectively, because of the kinds of distributions (incremental and differential, respectively) and their relationships with the average pore radius with respect to the pore volume or the SSA.

### 2.3. The Structure of the Electrical Double Layer (EDL)

#### 2.3.1. Surface Charge Density

The potentiometric titration of the suspension is the most commonly used method to determine the surface charge density and position of the zero point of charge (PZC) in the composite at the sparingly soluble/electrolyte solution system interface. The surface charge density and the point of zero charge are important parameters to characterize the electrical double layer (EDL) at the composite/electrolyte solution interface. These values, apart from the zeta potential and adsorption density of carrier electrolyte ions, are helpful in determining the structure of the EDL at the interface.

The charge on the surface of the composite is created as a result of the reaction between the hydroxyl groups on the surface of this composite and the components of the liquid phase. In the case of aqueous solutions, hydrogen ions (hydroxide) and ions of the carrier electrolyte play an important role in creating a charge at the interface with the composite. Hydrogen ions affect the accumulation of charge on the surface of the composite through the acid-base reactions of surface hydroxyl groups:

(2) $${\mathrm{SOH}}_{2}^{+}↔{\mathrm{OH}}^{0}{+H}^{+}$$

(3) $${\mathrm{SOH}}^{0}↔{\mathrm{SO}}^{-}+H^{+}$$

Therefore, hydrogen ions create a charge on the surface of the composite and are considered potential determining ions (PDIs) for these systems. In addition to hydrogen ions, the ions of the carrier electrolyte play an important role in generating the charge in these systems. In classical theories, these ions undergo non-specific adsorption, while in more complex models, the ions of the carrier electrolyte can also undergo specific adsorption. According to the site binding theory, characteristic of the oxide systems, the ions of the carrier electrolyte react with the surface hydroxyl groups of the oxide to form complex connections, the following complexation reactions can take place on the composite surface:

(4) $${\mathrm{SOH}}_{2}^{+}{\mathrm{An}}^{-}↔{\mathrm{SOH}}^{0}+H^{+}+{\mathrm{An}}^{-}$$

(5) $${\mathrm{SOH}}^{0}+{\mathrm{Ct}}^{+}↔{\mathrm{SO}}^{-}{\mathrm{Ct}}^{+}+H^{+}$$

These connections, together with the forms created in reactions (1) and (2), determine the charge on the adsorbent surface. Anions (An) reacting with the surface hydroxyl groups, according to reaction (4), cause the formation of a positive charge on the surface of the adsorbent, and they locate themselves in the compact layer of the double electrical layer in the so-called Inner Helmholtz Plane (IHP—Inner Helmholtz Plane). On the other hand, cations (Ct) of the basic electrolyte adsorb and contribute to the increase of the negative charge on the surface of the adsorbent and, similarly to anions, compensate for this charge by occupying a position in the Helmholtz plane. In most EDL models, the simplification of assigning cations and anions to the same plane is used.

The surface charge density is the algebraic sum of the charges of the groups resulting from reactions (2)–(5):

$$\sigma_{0}=B*\left\{\equiv {\mathrm{SOH}}_{2}^{+}+\equiv {\mathrm{SOH}}_{2}^{+}{\mathrm{An}}^{-}-\equiv {\mathrm{SO}}^{-}{\mathrm{Ct}}^{+}-\equiv {\mathrm{SO}}^{-}\right\}$$

where *B*—the conversion factor of the surface concentration expressed in μmol/m^2^ into the charge density expressed in μC/cm^2^.

The structure of the electrical double layer formed at the solid–liquid interface determines the stability properties of suspensions, which is particularly important in the case of applying obtained composites in cosmetics. The specific position of the adsorbed molecules can be characterized by the sign and magnitude of the surface charge density (*σ*_0_) and the zeta potential (ζ). pH_IEP_ represents the situation where the zeta potential is 0 (concentrations of positively and negatively charged surface groups are the same). Negatively charged groups and ions in the area of the slip plane are the same. The surface charge is a physicochemical property that is associated with the composition of the composites.

**Figure 3:**
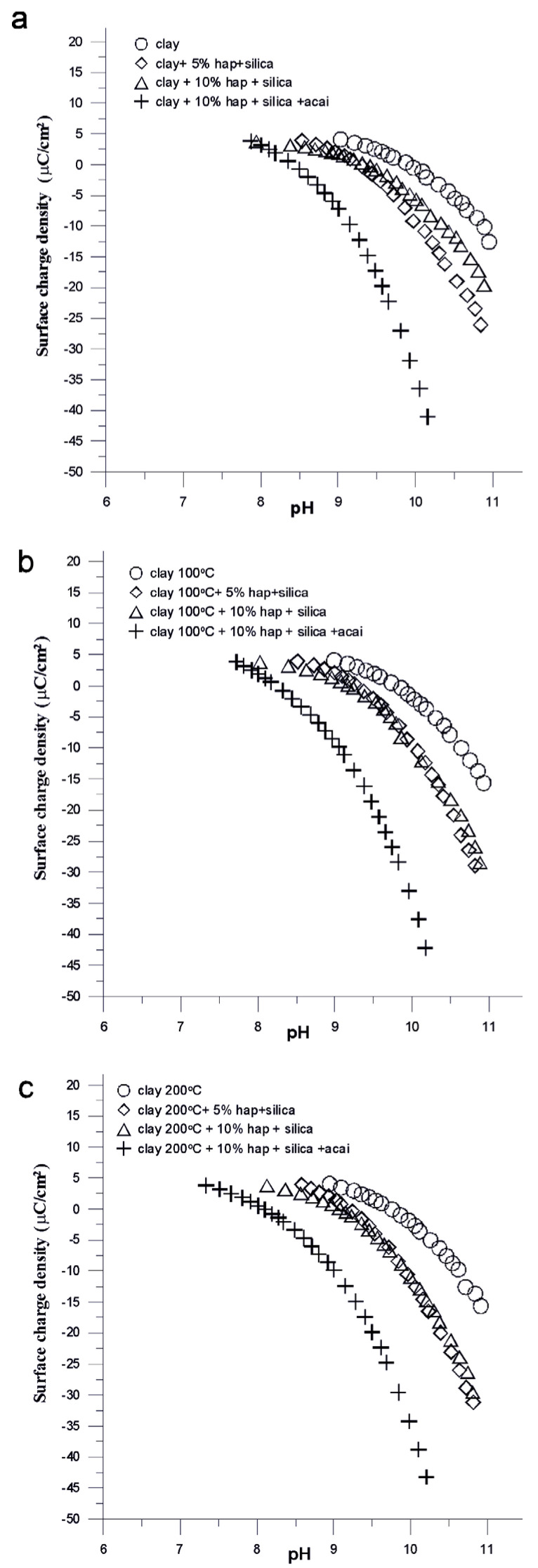
Surface charge density as a function of pH for the samples (**a**) s11, s1, s4, and s9; (**b**) s12, s2, and s5; and (**c**) s13, s3, s6, and s10 all dispersed in the NaNO_3_ solution.

Figure 3 shows the dependence of the surface charge density on pH for selected composites. In all cases, the measurements were performed in the pH range from 7 to 11, i.e., in which the individual components of the composites do not dissolve. In Figure 3a–c, one can see that the surface charge density decreases with increasing pH, which is the result of the reactions taking place on the surface of the selected composites. As follows from Table 2, there is a clear shift in the pH_pzc_ point. For pure clay heated at different temperatures, there is a shift from 9.9 to 9.6, which may be due to a change in the structure of the pore surface. If the samples of pure clay and its composites are taken into account, a clear shift can be seen in pH_pzc_ in the less alkaline direction that may be the result of some functionalities appearing on the surface of the composite responsible for creating a more negative charge. The same tendency for both unheated and preheated (at 100 °C and 200 °C) clay composites can be seen.

#### 2.3.2. Zeta Potential

One of the basic experimental parameters describing the structure of the electrical double layer formed at the solid/solution interface is the zeta potential, usually calculated from the electrophoretic mobility. The value of the zeta potential describes the mutual interactions of electrons at the interface in the dispersion systems. Knowing the value of the zeta potential, it is possible to determine the causes of flocculation and aggregation of particles in a given colloidal system, owing to which, for example, the stability of a given colloidal system can be improved.

In this experiment, the zeta potential was measured using an electrophoretic cell. The measurement consists in examining how the solid particles, in the case of this composite experiment, move in an electric field when a voltage is applied to the electrophoretic cell. The Smoluchowski equation presented below was used for the calculations:

$$u_{e}=\frac{\varepsilon_{0}\varepsilon \zeta }{\eta },$$

where *ε*—the relative permittivity of the medium, *ε*_0_—the electric permittivity of a vacuum, *η*—the viscosity of the liquid surrounding the particle, and *u_e_*—the voltage on the measuring cell.

**Figure 4:**
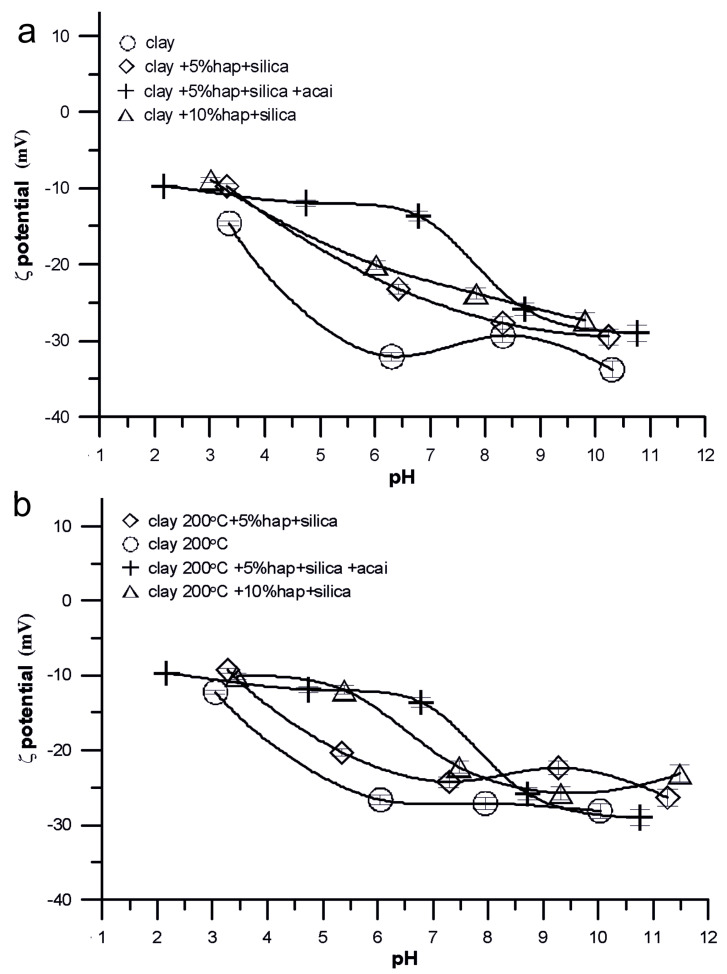
Dependence of zeta potential on pH for the composites: (**a**) clay, s1, s7, and s4; (**b**) clay 200 °C, s3, s8, and s6.

As shown in Figure 4, the tested systems are characterized by the highest absolute values of the zeta potential for the clay/electrolyte solution systems and the lowest values for the clay, 5% HA, and silica and acai systems. It was found that the zeta potential decreased with the increasing pH of the tested electrolyte. This may result from the dissociation of surfaces subjected to ionization on the selected composites. The state of the solid surface where the amounts of positive and negative charges in the dispersed electrical double layer are equal is called the Solid Surface IsoElectric Point (IEP), represented by pH_IEP_. As a result, the net charge of the dispersed layer of the electric double layer is zero. Because the concentration of the potential-forming ions (H^+^ and OH^−^) depends on the pH of the solution, the pH_IEP_ corresponds to the precise pH value. The pH_IEP_ values for all composites are below pH 2, which may affect the electrostatic interactions with other electrolyte particles surrounding the composites.

The tested samples in the composite/electrolyte solution system are colloidally unstable. The factors affecting the colloid stability are the size of the dispersed particles (preferably very small), the charge, and the presence of the solvation shell (hydrophilic colloids). The tested composites in the entire tested pH range had zeta potential values from −10 mV to −30 mV, stable systems are considered to be those where the zeta potential value is greater than −30 mV.

Differences in the pH_pzc_ and pH_iep_ values for the individual composites may result from the overlapping of the electrical double layers forming in the pores of the samples and may be caused by the attachment of negatively charged functionalities on the composite surface as a result of the adsorption of electrolyte ions.

In Figure 3, based on the dependence of the surface charge density on pH and the zeta potential on pH, one can see similar tendencies between the pure clay sample and the composites consisting of clay, HA, silica, and acai of being very different from each other and the samples without acai with a variable composition of composites are very similar, they take intermediate values for both surface charge density and zeta potential.

### 2.4. Particles Size

All tested composites were composed of nanometric sizes particles, but the average sizes (Table 3) correspond to the nanoparticles aggregates. Comparing the results, one can see that annealing rose clay had an effect on the samples’ sizes. The higher the heating temperature of the initial clay, the smaller the particle size of the composites, which was probably due to the sintering of the pores in the clay. This phenomenon spontaneously occurs with an increase in temperature, the direction of which is determined by the decrease in free enthalpy and is accompanied by a decrease in the development of clay-free surfaces. The binding of grains is accompanied by shrinkage of the entire system and the transition of loose or poorly bound powder into a solid. All composites suspended in the seawater were smaller than 400 nm in size. Some of them were smaller than those in the deionized water.

### 2.5. Rana—Cancer, Developmental & Reproductive Toxicity, Allergies & Immunotoxicity

The level of safety of the obtained materials and plant raw materials was assessed (Table 4) using the program “Rana” written by V. Paientko. All composites of clay/hydroxyapatite, clay/silica, and clay/hydroxyapatite/silica were not higher than 21 and were hypoallergenic. The samples, with the addition of vegetable raw materials, are hypoallergenic too. Based on the obtained results, it can be concluded that all obtained composites can be used as additives to cosmetics.

### 2.6. Tests on the Release of Acai

The release of anthocyanins from the composite materials was investigated (Table 5). The effect of heating the initial clay on the isolation of anthocyanins from hybrid materials was also studied.

The composition of the hybrid material and the heating of the starting clay affect the release of anthocyanins.

Table 5 shows that the composites, particularly s7, s9, rose clay (100 °C)/Hydroxyapatite (10%)/silica/acai, and s8, are better nanostructured composites that are more active in releasing anthocyanins (maximum for s8). It should be noted that more bioactive composites such as s7, s8, and s9 have practically minimal *S*_BET_ and *V*_p_ values (Table 2). This is rather an apparent contradiction because the low porosity of rose clay and composites based on it causes the localization of acai plant raw materials from pores (or in the macro-sized voids), which can provide better conditions for the bioactive compounds released from poorly bound surfaces. From the spaces, which are less restrictive, plant raw materials of acai are more readily released.

The structure of the obtained composites based on rose clay confirms that it transforms into a better nanostructured state and the minimally limited spatial effects in the meso/macroporous composites can be significant to improve the release of bioactive compounds, which can be largely increased compared with those of plant raw materials, i.e., acai alone.

## 3. Methods and Materials

The samples of rose clay/hydroxyapatite/silica/vegetable raw materials were prepared using the method of mechanochemical activation (Table 6). Rose clay was purchased from “Mel-OK”, Kyiv, Ukraine. In the tests, a part of clay was annealed at temperatures of 100 °C and 200 °C. Hydroxyapatite was prepared using the wet method at the Department of Radiochemistry and Environmental Chemistry, UMCS (Lublin, Poland). After the synthesis, it was washed to obtain the proper water conductivity over the sediment of 1 μS. Fumed silica was synthesized at the pilot plant of the Institute of Surface Chemistry (Kyiv, Ukraine). A solution of the seawater (by Beurre) with the purity of pure d.a. was used for the tests. Seawater is a solution of sodium chloride (NaCl), with a concentration of 0.9 to 3%. The most characteristic feature of seawater is the large concentration of cations (Na^+^, K^+^, Mg^2+^, and Ca^2+^) and anions (Cl^−^, HSO_4_^−^, SO_2_^−4^, HCO_3_^−^, and CO_2_^−3^), which makes intensely bitter or bitter-salty tasting (undrinkable) seawater. The other electrolyte taken for testing was 0.001 mol/dm^3^ of pure NaNO_3_ from Sigma-Aldrich (St. Louis, MO, USA).

The powder XRD patterns of rose clay–hydroxyapatite–silica composites were recorded using the DRON-4–07 diffractometer (“Burevestnik”, Burevestnik, Russia) with the Cu K_α_ radiation (λ = 0.15418 nm), and the X-ray beam focusing on the Bragg–Brentano geometry in the angular range of 5–80 degrees. The identification of the phases was made using the X-ray database PDF-2.

To estimate the textural characteristics of the samples degassed at 383 K for 12 h (Table 1), low-temperature (77.4 K) nitrogen adsorption–desorption isotherms were recorded using the Micromeritics ASAP 2460 adsorption analyzer. The specific surface area (Table 1, *S_BET_*) was calculated according to the BET method [26] at *p*/*p*_0_ between 0.06 and 0.2 (where *p* and *p*_0_ denote the equilibrium and saturation pressures of nitrogen at 77.4 K, respectively). The total pore volume (Table 1, *V*_p_) was evaluated from the nitrogen adsorption at *p*/*p*_0_ ≈ 0.98–0.99 [27]. The nitrogen desorption data were used to compute the pore size distributions (PSD, differential *f*_V_(*R*)~d*V*_p_/d*R,* and *f*_S_(*R*)~d*S*/d*R*) using the self-consistent regularization (SCR) procedure under the non-negativity condition (*f*_V_(*R*) ≥ 0 at any pore radius *R*) at the fixed regularization parameter of α = 0.01. The complex pore model was applied with the slit-shaped (S) and cylindrical (C) pores in silica and voids (V) between the spherical nanoparticles packed in the random aggregates (SCV/SCR method) [28,29]. For the pure clay samples, the SC/SCR model (with the slit-shaped and cylindrical pores) was used. The differential PSD, with respect to the pore volume of *f*_V_(*R*) ~ d*V*/d*R*, ʃ*f*_V_(*R*)d*R*~*V*_p,_ was re-calculated to the incremental PSD (IPSD) at Φ_V_(*R_i_*) = (*f*_V_(*R_i_*_+1_) + *f*_V_(*R_i_*))(*R*_i+1_ − *R*_i_)/2 at ∑Φ_V_(*R_i_*) = *V*_p_. The *f*_V_(*R*) and *f*_S_(*R*) functions were also used to calculate the contributions of nanopores (*V*_nano_ and *S*_nano_ with the radius in the range of 0.35 nm < *R* < 1 nm), mesopores (*V*_meso_ and *S*_meso_ at 1 nm < *R* < 25 nm), and macropores (*V*_macro_ and *S*_macro_ at 25 nm < *R* < 100 nm). Additionally, the non-local density functional theory (NLDFT), Quantachrome software (version 4.0), and the model of cylindrical pores in silica method [30] were used to calculate the differential PSD. The average values of the pore radii <*R_X_*> could be determined with respect to the pore volume (X *=* V) and the specific surface area (X = S) as the ratio of the first and zero moments of the distribution functions.

(6) $$<R_{X}>=\int_{R_{\min}}^{R_{\max}}Rf_{X}(R)dR/\int_{R_{\min}}^{R_{\max}}f_{X}(R)dR$$

Additionally, *f*_S_(*R*) could be used to estimate the deviation (Δ*w*) of the pore shape from the model using [31,32]:

(7) $$\Delta w=S_{BET}/\int_{R_{\min}}^{R_{\max}}f_{S}(R)dR-1$$

where *R*_max_ and *R*_min_ are the maximal and minimal pore radii, respectively. The S^*^_nano_, S^*^_meso,_ and S^*^_macro_ values could be corrected by multiplication by (Δ*w* + 1), which gives S^*^(Δ*w*+ 1) = *S*_sum_ = *S*_nano_ + *S*_meso_ + *S*_macro_ = *S_BET_*. The effective *w* value (*w*_ef_) can be estimated with the equation

(8) $$w_{ef}=(S_{BET}/V_{p})\int_{R_{\min}}^{R_{\max}}Rf_{V}(R)dR/\int_{R_{\min}}^{R_{\max}}f_{V}(R)dR$$

However, the reliability of the Δ*w* value depends on the reliability of both *S*_BET_ and PSD.

The zeta potential was measured with the electrophoretic method using a Nano-ZS Zetasizer, Malvern. Measurements were made at 25 °C. In the zeta potential calculations, Smoluchowsky’s equation was also applied because of *κa*~150.

Solutions with the same parameters as the measurement of the surface tension were prepared in 50 cm^3^ flasks. They were tested with a NanoZS Zetasizer (Malvern Instruments, Malvern, UK). The measurements were performed at 100 ppm solid concentration of composites. The compound was added to the solution and subjected to dispersion using a Sonicator XL 2020 ultrasound probe. Then, the suspension was poured into 125 mL flasks and pH was established to be in the range of 4–11 using the 0.1 mol/dm^3^ HNO_3_ and NaOH solutions. Five measurements of zeta potential were made for each solution.

The surface charge density of the composites in the suspensions was determined using the potentiometric titration method. An amount of 0.001 mol/dm^3^ of NaNO_3_ was used as a background electrolyte. The composites were added into the thermostated vessel to the volume of 50 cm^3^, and the surface charge density of the composites’ suspension was estimated. All the studied suspensions were titrated with the 0.1 mol/dm^3^ NaOH solution in the pH range of 4–11. The surface charge densities of the studied samples were calculated with the titr_v3 program elaborated by W. Janusz (UMCS).

The particle size distribution was determined using the Mastersizer 2000 (Malvern, Pennsylvania, PA, USA). Measurements were made in redistilled water and seawater.

The release of anthocyanins from the plant raw materials and clay/hydroxyapatite/plant raw materials composites was studied using the *PerkinElmer* UV spectrophotometer (PerkinElmer, Waltham, MA, USA).

To assess the level of safety of the obtained materials, the software product “Rana”—the information system was used. It was designed to store and organize the composition data and calculate the development of cosmetics and other products or fillers, as well as determine their level of safety in terms of the component composition of the final mixture. Safety was assessed based on three indicators—cancer, developmental and reproductive toxicity, and allergies and immunotoxicity.

## 4. Conclusions

Based on the obtained results for a set of composites, it can be seen that the sample preparation method affects the particle size, surface charge density, zeta potential, and pH value of the aqueous suspensions. With the increasing preheating temperature of rose clay, the particle (aggregates) size can decrease, and the surface charge density changes as well as the zeta potential. This affects the release of bioactive compounds from the composites with acai.

Nanostructured composite blends based on rose clay—with the major crystalline phases of α-quartz and kaolinite with the addition of hydroxyapatite, nanosilica, and acai leaf powder (30 wt.%)—remain porous and similar to (or stronger than) the initial rose clay after the mechanical processing. The mixtures mainly retain the morphological and textural characteristics of the components due to the mechanical treatment at room temperature for the air-dry powders with a relatively low load. The amounts of water (adsorbed from the air) contained in the hydrophilic components can promote a smearing effect useful for the reorganization of nanostructured aggregates and agglomerates of aggregates. For most blends, the textural characteristics are greater than those of rose clay alone. The research confirms the positive effects of small additions of nanosilica and nanostructured hydroxyapatite (5–10 wt.%) in several aspects, including the bioactivity of the composites.

For the proposed composite materials of clay/nanosilica/vegetable raw materials/hydroxyapatite, the safety indicators of components and composite materials were studied. The possibility of their use in cosmetics is substantiated. The hypoallergenicity of all the studied systems is confirmed. Kinetic studies of BAS release (from acai in composites) can be used as a factor in regulating the direction of preventive action of cosmetics.

The release of cyanidin-3,5-diglycoside (anthocyanins) as a bioactive compound is greater for the composites than for acai with rose clay alone. The composites with clay/nanosilica/vegetable raw materials/hydroxyapatite can be considered better systems for cosmetics and medicinal preparations than rose clay alone because the control of morphological, electrochemical, and textural features of the composites allows one to ensure adequate activity of the systems.

## Figures and Tables

**Table 1:** Textural characteristics of initial the studied and modified rose clay samples (DFT SCV/SCR).

Sample	*S_BET_* (m^2^/g)	*S*_NLDFT_ (m^2^/g)	*V*_p_ (cm^3^/g)	*V*_nano_ (cm^3^/g)	*V*_meso_ (cm^3^/g)	*V*_macro_ (cm^3^/g)	*S*_nano_ (m^2^/g)	*S*_meso_ (m^2^/g)	*S*_macro_ (m^2^/g)	<*R*_V_> (nm)	<*R*_S_> (nm)	Δ*w*	*c*_slit_	*c*_cyl_	*c*_sph_
s1	59	58	0.144	0.022	0.097	0.024	36	22	1	13.9	2.7	0.056	0.892	0.101	0.007
s2	45	42	0.125	0.016	0.083	0.026	26	18	1	16.3	3.1	0.044	0.861	0.132	0.007
s3	43	39	0.117	0.016	0.083	0.017	25	18	1	12.8	2.8	0.016	0.910	0.076	0.014
s4	58	57	0.136	0.021	0.092	0.023	35	21	1	14.4	2.7	0.077	0.886	0.095	0.019
s5	35	30	0.125	0.012	0.089	0.024	18	17	1	16.7	3.8	−0.011	0.836	0.120	0.044
s6	45	41	0.134	0.014	0.088	0.031	24	20	1	18.5	3.6	0.135	0.771	0.053	0.176
s7	19	17	0.080	0.007	0.057	0.016	9	10	0	16.8	4.1	−0.066	0.900	0.073	0.027
s8	18	16	0.082	0.006	0.055	0.021	8	10	1	20.1	4.7	0.0	0.859	0.093	0.048
s9	22	20	0.099	0.008	0.072	0.019	10	12	1	16.1	4.2	−0.088	0.866	0.094	0.040
s10	22	19	0.101	0.008	0.075	0.018	9	12	0	16.7	4.2	−0.056	0.944	0.037	0.019
s11	51	55	0.088	0.020	0.053	0.015	35	16	1	11.9	2.0	0.081	0.880	0.120	-
s12	37	38	0.087	0.015	0.057	0.015	24	13	0	13.2	2.5	0.002	0.884	0.116	-
s13	37	37	0.090	0.014	0.060	0.015	22	14	1	13.5	2.6	0.033	0.899	0.101	-

Note. The first and second values in *S*_NLDFT_ correspond to a model with cylindrical pores in silica. The *V*_nano_ and *S*_nano_ values were calculated by integration of the *f*_V_(*R*) and *f*_S_(*R*) function, respectively, at 0.35 nm < *R* < 1 nm, *V*_meso_ and *S*_meso_ at 1 nm < *R* < 25 nm, and *V*_macro_ and *S*_macro_ at 25 nm < *R* < 100 nm. Equation (6) is the average pore radii with respect to the pore volume (X = V) and specific surface area (X = S). $\Delta w=(S_{BET}/\int_{R_{\min}}^{R_{\max}}f_{S}(R)dR-1)\times 100$ is the DFT SCV/SCR model error. The values of *c*_slit_, *c*_cyl_, and *c*_sph_ correspond to contributions of slit-shaped and cylindrical pores and voids between spherical particles.

**Table 2:** pHpzc values for the tested composites.

Sample	Compositions	Native Clay	100 °C Clay	200 °C Clay
		pH_pzc_		
s11, s12, s13	Clay	9.9	9.8	9.6
s1, s2, s3	Clay, 5% HA, silica	9.4	9.3	9.1
s4, s5, s6	Clay, 10% HA, silica	9.2	9.2	9.1
s9, s10	Clay, 10% HA, silica, acai	8.4	8.2	8

**Table 3:** Particle sizes in water and sea water for the samples.

No.	Samples		Average Particle Size [nm]	
Component Contents			Water	Sea Water (Electrolyte)
1	s11	Clay	544.1	279.8
2	s12	Clay 100 °C	368.1	341.0
3	s13	Clay 200 °C	354.9	362.8
4	s1	Clay + 5% HA + silica	488.3	393.0
5	s2	Clay 100 °C + 5% HA + silica	369.1	377.4
6	s3	Clay 200 °C + 5% HA + silica	347.9	334.9
7	s4	Clay + 10% HA + silica	467.5	345.1
8	s5	Clay 100 °C + 10% HA + silica	380.0	339.4
9	s6	Clay 200 °C + 10% HA + silica	347.9	335.2
10	s9	Clay + 10% HA + silica+ acai	359.0	389.0
11	-	Clay 100 °C + 10% HA + silica+ acai	335.9	343.6
12	s10	Clay 200 °C + 10% HA + silica +acai	324.6	375.8

**Table 4:** Assessment of the level of safety of composite materials and vegetable raw materials.

#	Sample	Component Contents	Cancer	Developmental & Reproductive Toxicity	Allergies & Immunotoxicity
1	-	Hydroxyapatite (HA)	18	18	20
2	-	Silica (A300)	20	18	21
3	-	Acai (euterpe oleraceae (acai) berry)	18	18	21
4	-	Rose clay/HA/Acai	19	18	20
5	-	Rose clay/Silica/Acai	19	18	21
6	s7	Rose clay/HA/Silica/Acai	19	18	21
7	-	Rose clay/HA	20	19	20
8	-	Rose clay/Silica	21	19	21
9	s1	Rose clay/HA/Silica	20	18	20

**Table 5:** Release of anthocyanins from the hybrid composites and plant raw materials.

No	Sample	Component Contents	A (a.u.)	C (mg/g)	C (µg/g)
Native clay					
1	-	Rose clay/Acai	0.050	0.30	30.1
2	-	Rose clay/HA (5%)/Acai	0.052	0.030	30.21
3	-	Rose clay/Silica/Acai	0.057	0.033	33.11
4	s7	Rose clay/HA (5%)/Silica/Acai	0.065	0.038	37.76
5	-	Rose clay/HA (10%)/Acai	0.045	0.032	31.88
6	s9	Rose clay/HA (10%)/Silica/Acai	0.059	0.043	42.84
Rose clay,100 °C					
7	-	Rose clay/Acai	0.056	0.033	32.53
8	-	Rose clay/HA (5%)/Acai	0.057	0.033	33.11
9	-	Rose clay/Silica/Acai	0.054	0.031	31.37
10	-	Rose clay/HA (5%)/Silica/Acai	0.049	0.029	29.35
11	-	Rose clay/HA (10%)/Acai	0.057	0.033	33.11
12	-	Rose clay/HA (10%)/Silica/Acai	0.062	0.042	41.88
Rose clay, 200 °C					
13	-	Rose clay/Acai	0.068	0.049	49.38
14	-	Rose clay/HA (5%)/Acai	0.073	0.042	42.41
15	-	Rose clay/Silica/Acai	0.061	0.055	54.52
16	s8	Rose clay/HA (5%)/Silica/Acai	0.071	0.082	82.49
17	-	Rose clay/HA (10%)/Acai	0.091	0.068	67.77
18	s10	Rose clay/HA (10%)/Silica/Acai	0.078	0.045	45.31

**Table 6:** Composition of the studied samples and preheating temperature (T).

Sample	Rose Clay (wt.%)	Hydroxyapatite (HA) (wt.%)	Nanosilica (wt.%)	Acai (wt.%)	Preheating of Clay (°C)
s1	92	5	3	0	-
s2	92	5	3	0	100
s3	92	5	3	0	200
s4	87	10	3	0	-
s5	87	10	3	0	100
s6	87	10	3	0	200
s7	62	5	3	30	-
s8	62	5	3	30	200
s9	57	10	3	30	-
s10	57	10	3	30	200
s11	100	0	0	0	-
s12	100	0	0	0	100
s13	100	0	0	0	200

## Data Availability

Not applicable.
